# Early cardiac proteomic profiling in a long-latency doxorubicin cardiomyopathy model exhibits sexual dimorphism for cardioprotection

**DOI:** 10.1016/j.jmccpl.2026.100861

**Published:** 2026-08-15

**Authors:** Sogol Sedighi, Lijun Chen, Yuefen Wang, Kaui Lebarbenchon, Jena Yi, Tanvi Menghani, Firasat Ali Shah, Sam Yang, Harumi Saeki, Tomoaki Ito, Hajime Orita, Hui Zhang, Brian O’Rourke, D. Brian Foster, Kathleen L. Gabrielson

**Affiliations:** a Division of Cardiology, Department of Medicine, Johns Hopkins University School of Medicine, Baltimore, MD, USA; b Department of Pathology, Clinical Chemistry Division, School of Medicine, Johns Hopkins University, Baltimore, MD, USA; c Department of Molecular and Comparative Pathobiology, Johns Hopkins University School of Medicine, Baltimore, MD, USA; d Department of Human Pathology, Juntendo University, Faculty of Medicine Tokyo, Japan; e Department of Surgery, Juntendo University Shizuoka Hospital, Shizuoka, Japan; f Department of Gastroenterology and Minimally Invasive Surgery, Juntendo University, Tokyo, Japan; g Department of Oncology, Johns Hopkins University School of Medicine, Baltimore, MD, USA

**Keywords:** Proteomics, Data-independent acquisition, DIA, Doxorubicin, Cardiomyopathy, Long latency, Female, Male

## Abstract

Anthracycline-induced cardiotoxicity remains a major limitation in cancer therapy, with substantial inter-individual variability in susceptibility, including documented sex differences. However, the molecular basis underlying these disparities remains incompletely understood. In this study, we employed large-scale data-independent acquisition proteomics in a long-latency rat model of doxorubicin-induced cardiomyopathy to characterize early cardiac proteomic remodeling. Longitudinal echocardiography demonstrated a significantly greater decline in fractional shortening in males compared to females. Early proteomic profiling identified 5184 proteins and revealed widespread remodeling across metabolic, mitochondrial, cytoskeletal, and stress-response pathways. While doxorubicin induced broad proteomic changes in both sexes, two-way limma-based sex-stratified analyses identified 430 proteins exhibiting significant sex-by-treatment interactions. Our results suggested that doxorubicin was associated with alterations in oxidative phosphorylation, fatty acid metabolism, and calcium handling pathways. A female-specific upregulation of mitochondrial proteins, including Sco1, Cox17, and Atp2a2, which may be associated with preserved energetics and Ca^2+^ handling, was detected. In contrast, male hearts demonstrated reduced Micu1 and RyR2 together with increased levels of Ppp1ca, findings that may indicate alterations in mitochondrial function and, excitation–contraction coupling. We also observed lower levels of calcineurin (Ppp3cb) in females. Baseline proteomic differences between sexes further implied distinct regulatory states that may shape the cardiac response to doxorubicin.Together, these findings suggest early proteomic remodeling upon doxorubicin treatment and represent candidate molecular targets that warrant functional validation in future studies. This work offers hypothesis-generating mechanistic insights into sex-specific responses to anthracycline-induced cardiomyopathy and underscores the importance of incorporating sex as a biological variable in cardio-oncology research.

## Highlights

• Early proteomic profiling reveals remodeling in latent Doxorubicin cardiotoxicity.
• Males show earlier, more severe functional decline than females.
• Doxorubicin drives mitochondrial, metabolic, and Ca^2+^ handling disruption.
• Male hearts show mitochondrial failure and impaired EC coupling.
• Female hearts activate adaptive mitochondrial cardio protection.

## Graphical abstract

**Figure:**
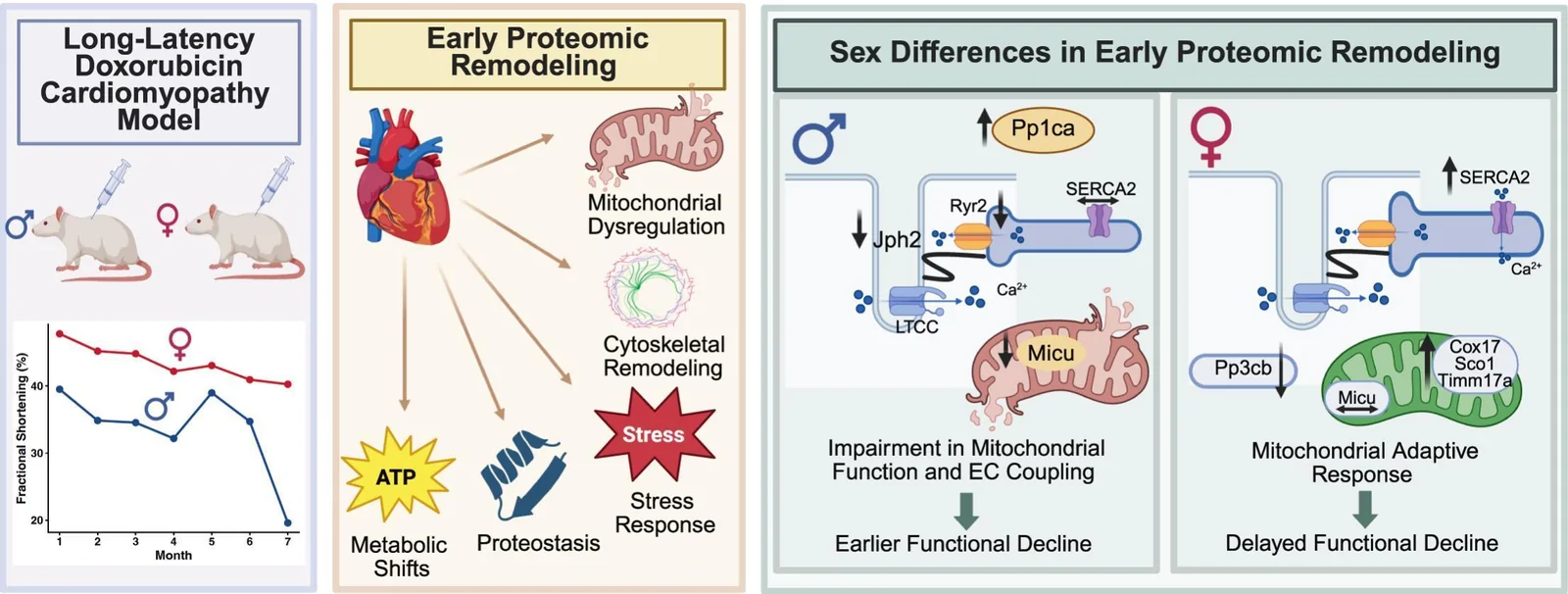

## 1 Introduction

Anthracyclines represent a longstanding and highly effective class of anticancer agents [1]. Annually, anthracycline-based treatment protocols are administered to approximately one million patients across North America, highlighting their continued significance in cancer therapeutics [1]. Anthracyclines are utilized in nearly 50% of breast cancer regimens and two-thirds of pediatric oncology protocols, contributing significantly to improved prognostic outcomes [2]. Agents like doxorubicin continue to serve as basis therapies for numerous malignancies, including hematological cancers (such as leukemia and lymphoma) and solid neoplasms (including breast, ovarian, uterine, and prostate carcinomas) [3]. Doxorubicin suppresses tumor growth by disrupting DNA replication and transcription. It impairs DNA replication through several complementary mechanisms, including intercalation into DNA, inhibition of DNA unwinding and strand separation via interference with helicase activity, eviction of histones from open chromatin, and inhibition of topoisomerase II [2]. However, with increased clinical implementation, a dose-dependent cardiac toxicity was recognized [1]. The risk of doxorubicin-induced cardiomyopathy is strongly dose dependent, rising to approximately 36% at cumulative doses above 600 mg/m^2^, with heart failure reported in about 26% of patients receiving more than 550 mg/m^2^ [2].

Doxorubicin-Induced cardiotoxicity (DIC) involves multiple biological pathways including: Ferroptosis, apoptosis, autophagy, energy metabolism disorders, immune responses, inflammation, oxidative stress and mitochondrial disorders [4]. Topoisomerase IIβ (Top2β) is a central mediator of doxorubicin-induced cardiotoxicity. In cardiomyocytes, doxorubicin binding to Top2β causes DNA double-strand breaks and prevents ligation, activates p53 signaling, and promotes apoptotic cell death, while simultaneously suppressing transcription of genes required for antioxidant defense and mitochondrial biogenesis, including PGC-1α and PGC-1β [5]. Early events in DIC include disruption of intracellular Ca^2+^ homeostasis, particularly depletion of sarcoplasmic reticulum Ca^2+^, which contributes to Ca^2+^/calmodulin-dependent kinase II dysregulation and promotes cardiomyocyte apoptosis [6]. At the metabolic level, doxorubicin disrupts fatty-acid β-oxidation (FAO) and tricarboxylic acid (TCA) cycle function, resulting in diminished ATP synthesis, elevated mitochondrial reactive oxygen species (ROS) production, and progressive cardiac damage [4]. In addition to mitochondrial DNA damage, doxorubicin compromises electron-transport-chain (ETC) complexes I, II, and IV, along with NADH dehydrogenase and cytochrome C oxidase, thereby perturbing mitochondrial permeability and bioenergetic equilibrium [7]. Doxorubicin induces profibrotic remodeling of the cardiac extracellular matrix through TGF-β– and cytokine-mediated pathways, contributing to myocardial stiffening and diastolic dysfunction. [8]

Notably, previous clinical and preclinical studies have suggested sex differences as a relevant risk factor for DIC [3]. The relationship between sex and anthracycline cardiotoxicity appears to be age-dependent; prepubertal females may demonstrate heightened susceptibility, whereas adult males appear to be at greater risk, while elderly males and postmenopausal women show more comparable risk profiles. In pediatric patients, female sex has been reported as an independent risk factor for anthracycline-related cardiac complications, with girls showing higher rates of cardiac dysfunction even after accounting for cumulative dose and other variables. In contrast, among adult patients, males appear to experience more severe cardiotoxicity, a pattern that is broadly consistent with findings in adult preclinical models. [9], [10].

In preclinical settings, experimental studies support sex as a determinant of disparity in doxorubicin-induced cardiotoxicity; male rodents treated with doxorubicin exhibit more severe myocardial lesions [3], mitochondrial dysfunction, and impaired calcium handling [11]. Increased fibrosis after doxorubicin exposure in males has been reported, partly due to inactivation of protective pathways such as apelin-APJ [12], whereas experimental studies suggest that female cardiomyocytes may manage mitochondrial activity and calcium signaling more effectively, and estrogen reduces oxidative modifications and preserves myofilament function, whereas testosterone does not [11], [12], [13], as ovariectomized female mice show greater injury than intact females [3].

Together, these clinical and experimental findings indicate that sex-specific vulnerability to doxorubicin cardiotoxicity arises from various biological processes. However, the molecular mechanisms driving these differences remain poorly understood. There are published proteomic datasets from rat models of doxorubicin cardiotoxicity that used doxorubicin doses that cause toxicity in weeks versus months [14], but none clearly present a comprehensive proteomics analysis comparing male and female rats within the same study cohorts.

To address this gap, we applied large-scale data-independent acquisition (DIA) proteomics, identifying 5184 proteins, in a rat model of doxorubicin-induced cardiomyopathy with extended latency, to characterize molecular changes that occur just after anthracycline exposure before heart function declines dramatically. Using a two-way limma framework to formally test sex-by-treatment interactions, this approach provided a broad view of early proteomic remodeling across key cardiac processes, including mitochondrial function, metabolism, contractile machinery, and stress-response pathways. By combining sex-stratified differential expression with pathway- and network-level analyses, we assessed how male and female hearts differ both at baseline and in their response to doxorubicin. Rather than identifying entirely distinct pathways, our analyses reveal sex-dependent differences in the magnitude of protein responses within shared cardiotoxic networks. We discuss the implications and prospects for functional validation.

## 2 Methods

### 2.1 Animal model

We developed a longer latency doxorubicin cardiomyopathy model with monthly systolic function measured by percent fractional shortening (FS) that declines over 6 or more months (versus weeks) in a rat model. Sprague-Dawley (SD) male and female rats (4–5 weeks of age) were randomly assigned to a cumulative doxorubicin (6 mg/kg) dose or an equal volume of saline treatment given by jugular vein injections under isoflurane anesthesia. The cumulative dose selected was significantly reduced from the cumulative dose that we had initially used in multiple studies (15 mg/kg) to induce systolic dysfunction in a shorter timeframe of 10–12 weeks from first doxorubicin injection [15], [16], [17]. With this lower dose, we expected a longer latency to the development of cardiac dysfunction. We used monthly transthoracic echocardiography to monitor rats who were habituated to restraint bags and were awake during a 4-min imaging session. When rats reached a decline in fractional shortening (FS) threshold based on our ACUC protocol (FS% < 30%), they were euthanized by CO_2_ and necropsied, with hearts fixed in formalin for histopathology analysis.

In parallel studies, using this same dosing model, for the proteomic studies, Sprague-Dawley (SD) male and female rats were randomly assigned to doxorubicin (6 mg/kg cumulative dose, 2 doses of 3 mg/kg, 2 weeks apart) or saline administered to rats by jugular injection at 4–5 weeks of age. One week after the last injection, rats were euthanized with CO2 and the whole hearts collected, frozen and cryo-pulverized for proteomic studies. Three biologically independent animals per group (Male Saline, Male Doxorubicin, Female Saline, Female Doxorubicin; *n* = 3 per group) were included in proteomic analyses.

### 2.2 Sample processing for protein extraction and tryptic digestion

Tissue lysis and downstream sample preparation for proteomic analysis was carried out as previously described [18]. Approximately 50–100 mg of each of the cryo-pulverized heart tissues were homogenized separately in an appropriate volume of lysis buffer (8 M urea, 75 mM NaCl, 50 mM Tris, pH 8.0, 1 mM EDTA, 2 μg/mL aprotinin, 10 μg/mL leupeptin, 1 mM PMSF, 10 mM NaF, Phosphatase Inhibitor Cocktail 2 and Phosphatase Inhibitor Cocktail 3 (1:100 dilution), and 20 uM PUGNAc) by repeated vortexing. Lysates were clarified by centrifugation at 18,000 x*g* for 10 min at 4 °C, and protein concentrations were determined by BCA assay (Pierce). Lysates were diluted to a final concentration of 8 μg/uL with lysis buffer, and 800 μg of protein was reduced with 5 mM dithiothreitol (DTT) for 1 h at 37 °C, and subsequently alkylated with 10 mM iodoacetamide for 45 min at RT in the dark. Samples were diluted 1:3 with 50 mM Tris-HCl (pH 8.0) and subjected to proteolytic digestion with LysC (Wako Chemicals) at 1:50 enzyme-to-substrate ratio for 2 h at RT, followed by the addition of sequencing grade modified trypsin (Promega) at 1:50 enzyme-to-substrate ratio and overnight incubation at RT. The digested samples were then acidified with 50% formic acid (FA, Fisher Chemical) to ~pH 2. Tryptic peptides were desalted on reversed phase C18 SPE columns (Waters). An aliquot of about 30μg peptides of each sample was then cleaned using house-made 3-layers stage tips and dried using Speed-Vac (Thermo Scientific).

### 2.3 LC-MS/MS analysis for DIA

Indexed retention time (iRT) standard peptide mixture (iRT kit, Ki-3002, Biognosys) was added into each samples prior mass analysis. SWATH-MS Data independent acquisition (DIA) data were collected by mass spectrometer (Orbitrap Fusion™ Lumos™ Tribrid™, Thermo Fisher Scientific) coupled with EASY-nLC™ 1200 (Thermo Fisher Scientific). Equal amount of peptides (0.8 μg) were loaded and separated on the house-packed column (0.75 μm I.D. x 26.5 cm length packed with ReproSil-Pur 120 C18-AQ, 1.9 μm) by two linear gradient 1) 7–25% of solvent B (90% ACN, 0.1% formic acid, solvent A; 3% ACN, 0.1% formic acid) for 90 min, and 2) 25–30% of solvent B for 28 min at a flow rate of 200 nL/min and column temperature of 50 °C. The DIA parameter was consisted of MS1 scan (350–1650 of *m*/*z* range, 120 K of resolution, and 3E106 of AGC value), followed by 30 of data independent MS2 scan segments. The MS2 scans were acquired by 35% NCE from 30 of MS1 window with 30 K resolution and 3E106 of AGC value.

### 2.4 Spectral library generation and DIA data analysis

DIA data was searched against a project-specific *Rattus norvegicus* spectral library generated directly from the DIA data using directDIA (library-free) mode in Spectronaut, combining all 12 runs in a single search, and a Uniprot FASTA database (version 6.11.2018; 24,712 entries) using Spectronaut (versions 12.0.2, Biognosys), according to the following criteria; fragment ions were selected with from 300 to 1800 *m*/*z*, minimal relative intensity of b, y ions was set as 5%, with a minimum of 3 matching y-ions [19]. Briefly, retention time of peptide feature was predicted by dynamic iRT setting with 1 min of correction factor window [20]. The peptide precursor and proteins were filtered with 1% q-value which was estimated with mProphet [21]. For quantification, interference fragment ions based on interfering signals and isotopes were removed. Protein quantities were determined with sum of top 3 peptides (minimum 1) which were calculated by area under the extracted ion chromatography (XIC) of top 3 transitions (b, y ion only).

### 2.5 Statistical analysis

Spectral intensities were quantified and processed using a median-based normalization strategy adapted from previously described workflows [22], [23]. Briefly, protein abundance values were processed as follows: (1) log₂ transformation, (2) median-centering within each sample, (3) median-centering across samples. Differential protein abundance between experimental groups was assessed using a Bayesian statistical framework implemented in linear models for microarray data (limma) with multigroup comparisons [24], using a two-way design incorporating sex (male vs. female) and treatment (saline vs. doxorubicin) as independent factors. Pairwise contrasts were performed where indicated. Moderated *p*-values were adjusted for multiple testing using the positive false discovery rate (q-value) approach described by Storey [25], [26]. Functional association networks were created for differentially expressed proteins using the StringApp plugin [27] embedded within Cytoscape (v3.10.4) [28]. Ontologically distinct network modules were extracted using the Markov clustering (MCL) [29] option in the ClusterMaker2 (v2.3.4) [30] app, specifying an edge-weight cutoff (i.e. evidence threshold) of 0.6 and an inflation parameter of 4. Heat maps were generated using the ComplexHeatmap R package with hierarchical clustering based on Euclidean distance and complete linkage to visualize relative abundance patterns across samples. Principal component analysis (PCA) was performed using the PCAtools R package. All code used for normalization, statistical analysis, PCA, and heat map generation is publicly available at: https://github.com/Frostman300/dox-upload

## 3 Results

### 3.1 Long-latency doxorubicin cardiotoxicity animal model

**Fig. 1:**
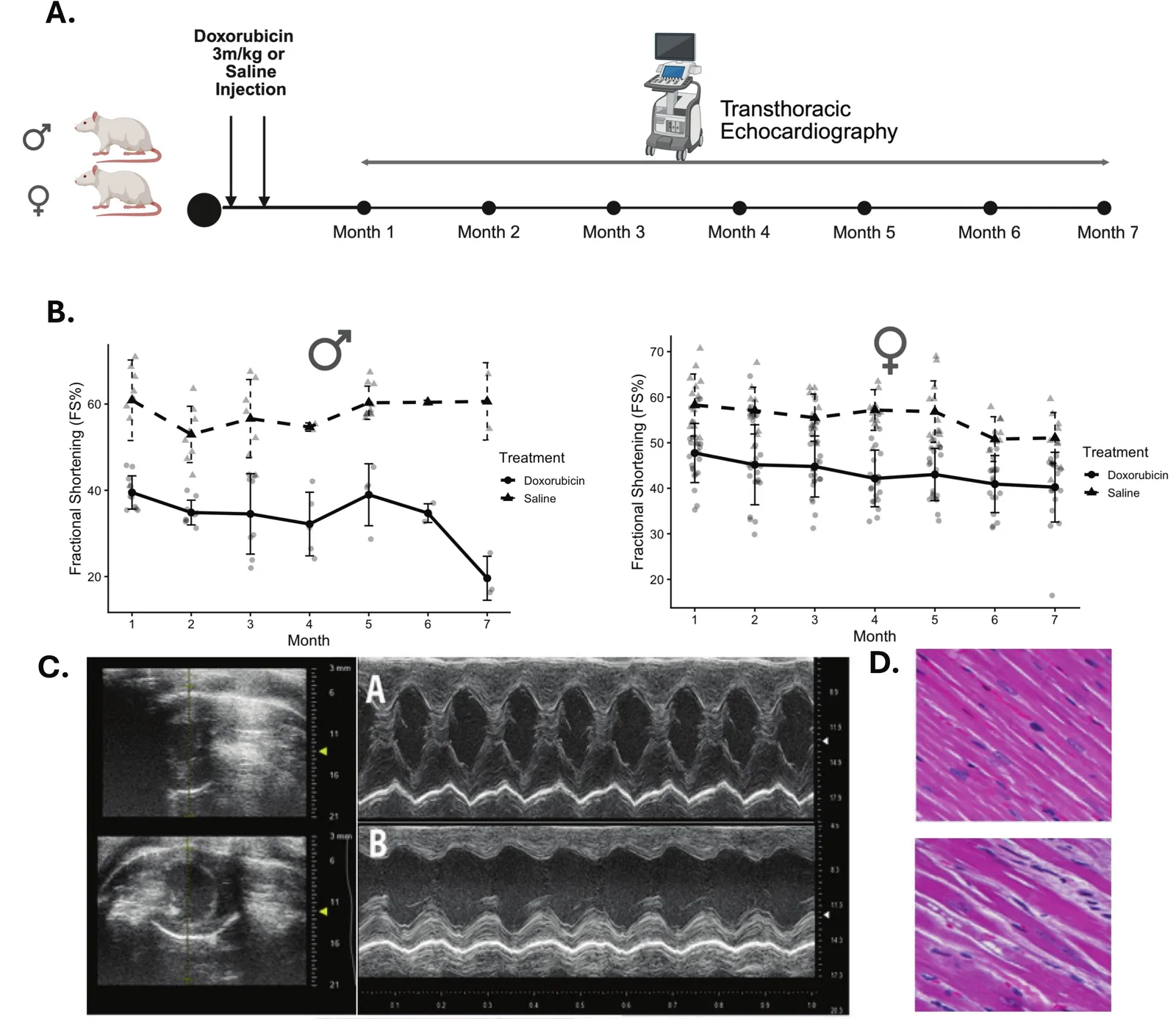
Longer latency Doxorubicin Cardiotoxicity Model (A) Experimental design-Created in BioRender: Male and female rats received a cumulative dose of 6 mg/kg doxorubicin or an equivalent volume of saline injection. Starting one month after the final injection, cardiac function was evaluated monthly by transthoracic echocardiography for up to 7 months. (B) Fractional shortening (FS) in saline- and doxorubicin-treated male rats (left) and female rats (right). Each data point represents an individual animal. Sample sizes per group vary across timepoints due to scheduled sacrifice for tissue collection; exact n per timepoint: Male Dox (*n* = 11, 9, 8, 5, 4, 3, 3), Male Saline (*n* = 8, 8, 7, 2, 10, 2, 2), Female Dox (*n* = 23, 22, 23, 22, 21, 17, 18), Female Saline (*n* = 14, 11, 13, 13, 12, 9, 9) for months 1–7 respectively. (C) Representative echocardiograms from conscious male rats showing left ventricular M-mode recordings (1-second duration) from saline controls (FS 62%, top) and doxorubicin-treated rats (FS 33%, bottom). (D) Representative histological sections of hearts from saline-treated (top) and doxorubicin-treated (bottom) male rats.

A schematic representation of the long-latency doxorubicin cardiotoxicity model is shown in Fig. 1A. As previously described, male and female rats received a cumulative dose of doxorubicin (6 mg/kg total; two injections of 3 mg/kg) or an equivalent volume of saline injection. Cardiac function was monitored by monthly transthoracic echocardiography in both male and female rats. Fractional shortening (FS) was measured longitudinally following treatment (Fig. 1B). Male rats exhibited a more pronounced decline in FS over time compared with females. A two-factor linear model considering treatment (doxorubicin vs. saline) and time (months) revealed a significant effect of treatment on FS in both males (*p* = 1.0 × 10^−9^) and females (*p* = 3.33 × 10^−6^). In doxorubicin-treated animals, FS declined significantly between month 1 and month 7 following the final doxorubicin injection in both male rats (39.5 ± 3.8 vs. 19.6 ± 5.1, *p* = 0.011) and female rats (47.7 ± 6.5 vs. 40.2 ± 7.7, *p* = 0.002), with the magnitude of functional decline approximately 2.6-fold greater in males than in females.

Representative 2D and M-mode echocardiographic images from control and doxorubicin-treated male rats at the terminal stage of the experiment are shown in Fig. 1C. According to our IACUC protocol, animals reaching a predefined FS decline threshold (FS < 30%) were euthanized by CO₂ and subjected to necropsy, with hearts fixed in formalin for histopathological analysis. Male rats were euthanized at 7 months or earlier post-chemotherapy to prevent unexpected mortality once FS had markedly declined, whereas female rats were euthanized if FS threshold was reached or at 12 months following doxorubicin or saline injection. Histologically, hearts from doxorubicin-treated rats demonstrated anecdotal interstitial fibrosis. (Fig. 1D; saline-treated male, top; doxorubicin-treated male, bottom), features consistent with those observed in patients with doxorubicin-induced cardiomyopathy. By 7 months following doxorubicin treatment, approximately 90% of male rats developed systolic dysfunction accompanied by cardiomyocyte degeneration and fibrosis. In contrast, female rats required a substantially longer period (approximately 12 months of follow-up) to develop comparable reductions in systolic function.

### 3.2 Global proteomic differences driven by doxorubicin treatment and sex

**Fig. 2:**
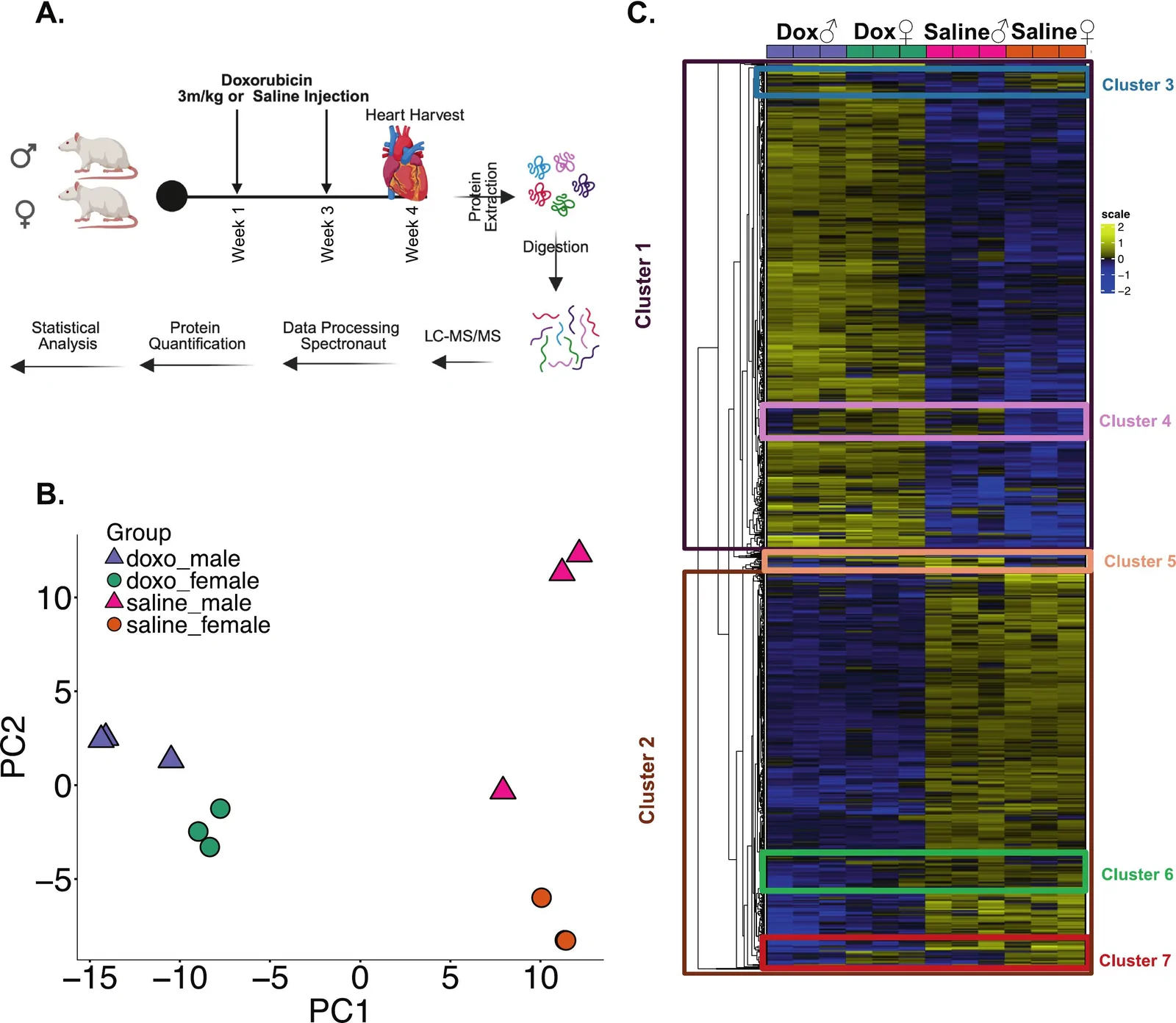
Global proteomic differences driven by doxorubicin treatment and sex*.* (A) Experimental design and proteomic analysis workflow-Created in BioRender (B) Principal component analysis (PCA) of proteins significant in the global F-test from a two-way limma model (*p* < 0.05) demonstrates clear separation between doxorubicin-treated and saline-treated hearts along PC1, indicating a dominant treatment-associated proteomic shift present in both sexes. Separation along PC2 reflects underlying sex-associated differences in the cardiac proteome.(C) Hierarchical clustering heatmap of the same protein set reveals distinct expression patterns across the proteome. *n* = 3 biologically independent animals per group (Male Saline, Male Doxorubicin, Female Saline, Female Doxorubicin). Each sample represents an individual rat heart.

In a parallel experiment using the same long-latency doxorubicin cardiotoxicity model, we assessed early proteomic remodeling by euthanizing animals one week after the final doxorubicin injection and processing hearts for downstream proteomic analysis (Fig. 2A). To comprehensively characterize the proteomic landscape, we performed a two-way limma analysis with sex and doxorubicin treatment as contributing factors. Principal component analysis (PCA) of proteins significant in the global F-test from a two-way limma model (*p* < 0.05) revealed that the majority of variance was determined by doxorubicin treatment. However, PC2 captured important sex-based differences that is present before and after doxorubicin treatment (Fig. 2B). Hierarchical clustering of same proteins demonstrated in PCA plot revealed seven distinct clusters with unique expression patterns (Fig. 2C). Clusters 1 and 2 captured the dominant proteomic response to doxorubicin, demonstrating broadly similar alterations in both sexes. Clusters 3, 5, and 7 highlighted sex-specific baseline differences in saline-treated hearts as well as differential responses between doxorubicin-treated males and females. Cluster 4 represented baseline proteomic differences between male and female hearts, while Cluster 6 specifically reflected sex-dependent difference upon doxorubicin treatment. Proteins comprising each cluster are shown in detail in Supplementary Fig. 1.

### 3.3 Impact of doxorubicin treatment on the cardiac proteome

**Fig. 3:**
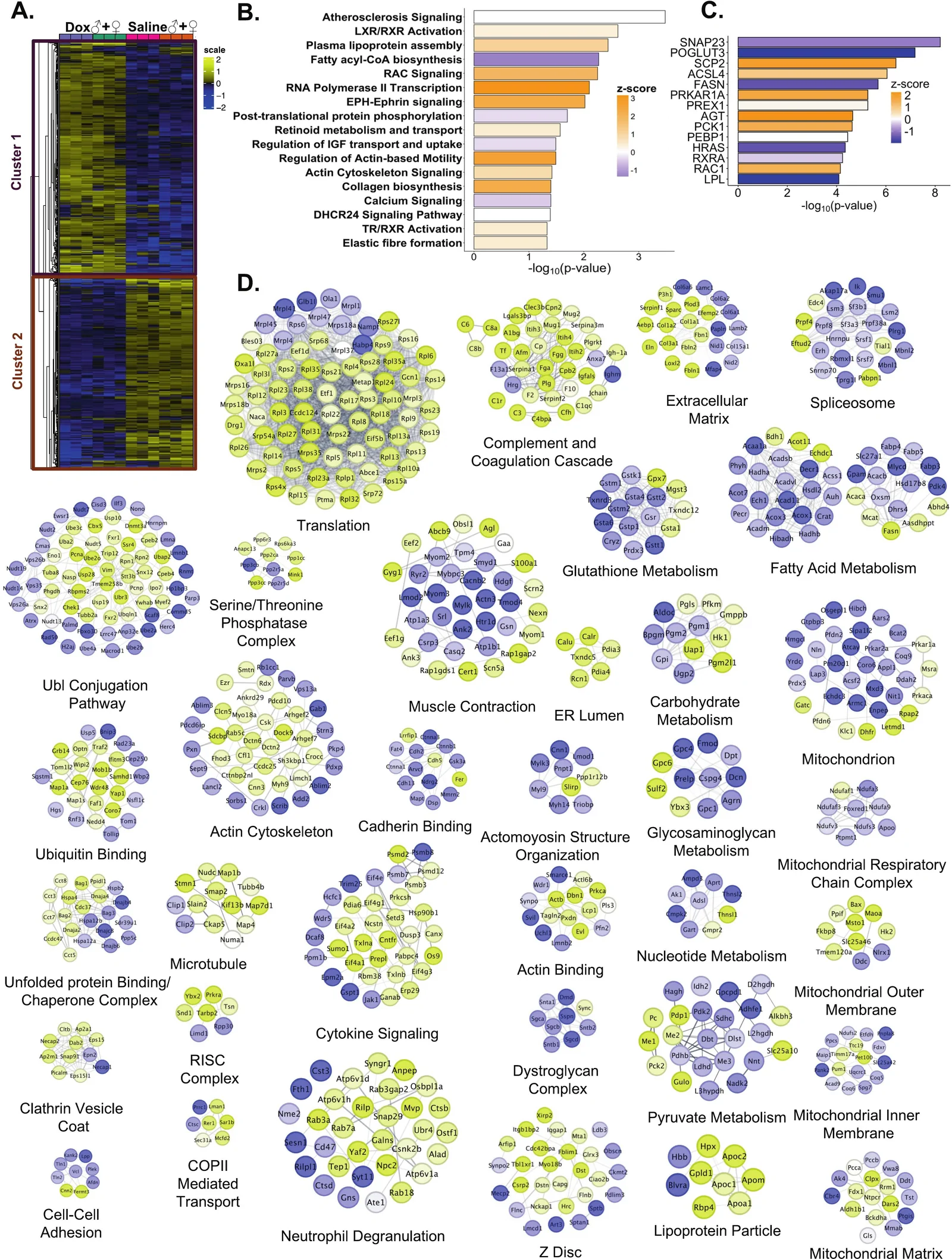
Impact of Doxorubicin Treatment on the Cardiac Proteome Independent of Sex. (A) Hierarchical clustering heatmap of proteins significantly altered by doxorubicin treatment (Doxo vs Saline, combined across females and males; two-way limma, *p*-value <0.05).Two major clusters (Clusters 1 and 2) capture the dominant proteomic response to doxorubicin. (B) Ingenuity Pathway Analysis (IPA) of these significantly altered proteins. Orange bars highlight pathways inferred to be activated, while blue bars represent pathways inferred to be inhibited (Fisher's exact test). (C) Predicted upstream regulators associated with the doxorubicin-induced proteomic changes. Orange indicates predicted activation, and blue indicates predicted inhibition compared to saline controls. (D) STRING network analysis of the same protein set, revealing coordinated enrichment of distinct functional modules. Blue nodes represent proteins downregulated in doxorubicin-treated hearts compared to saline controls, whereas yellow nodes represent upregulated proteins. *n* = 6 biologically independent animals per treatment group (Doxorubicin: 3 male +3 female; Saline: 3 male +3 female). (For interpretation of the references to colour in this figure legend, the reader is referred to the web version of this article.)

In analyses not stratified by sex, doxorubicin treatment significantly altered 1881 of 5184 detected proteins, highlighting its broad impact on the cardiac proteome. These proteins correspond to Clusters 1 and 2 identified in the hierarchical clustering heatmap **(**Fig. 2C**)** and were selected for downstream pathway analysis using Ingenuity Pathway Analysis (IPA). IPA revealed extensive remodeling of metabolic, signaling, and structural processes in the heart following doxorubicin exposure (Fisher's exact test). Pathways inferred to be activated (orange) included atherosclerosis signaling, LXR/RXR activation, Plasma lipoprotein handling, RAC signaling, RNA polymerase II–mediated transcription, and EPH–ephrin signaling. Additional pathways related retinoid metabolism, actin-based motility, and collagen biosynthesis were also significantly enriched, suggesting remodeling of proteostasis, extracellular matrix organization, and cellular stress responses. In contrast, pathways involved in fatty acyl-CoA metabolism, post translational phosphorylation, regulation of IGF transport, and calcium signaling were inferred to be inhibited (blue) (Fig. 3B). Upstream Regulator Analysis (URA) implicated several transcriptional and signaling drivers that would be consistent with the observed proteomic changes. SNAP23 and POGLUT3 showed the strongest predicted inhibition(blue). Additional affected regulators included lipid metabolic factors (ACSL4, FASN, RXRA), PKA signaling (PRKAR1A), and small GTPases (HRAS, RAC1). Additionally, LPL was predicted to be inhibited (blue), supporting disruption of lipid metabolism. (Fig. 3C).

Consistent with our pathway analysis, functional association network analysis conducted using STRING, and Markov-clustered for clarity, showed that doxorubicin-responsive proteins organized into multiple functional modules related to structural remodeling, mitochondrial function, signaling, and metabolism (Fig. 3D). Metabolic clusters were particularly prominent and included fatty acid, pyruvate, glutathione, and carbohydrate metabolism, suggesting extensive metabolic reprogramming in response to doxorubicin. Notably, proteins involved in fatty acid metabolism were predominantly downregulated, consistent with pathway-level findings. Similarly, glutathione and pyruvate metabolism showed an overall downward trend, whereas other metabolic pathways exhibited a mix of up- and downregulated proteins. Additional modules were enriched for mitochondrial organization and membrane components. Overall, we observed a broad downregulation of mitochondrial proteins, particularly those involved in the respiratory chain complexes, while proteins associated with the outer mitochondrial membrane tended to be upregulated(e.g Bax, Slc25a46,Fkbp8).

A prominent cluster of proteins involved in translation was identified, with most showing an upregulated pattern. Several networks also reflected substantial changes in protein turnover, including modules related to protein folding, ubiquitin-mediated conjugation, and spliceosome function. Cytoskeletal and structural remodeling was evident through enrichment of networks involving actin binding, microtubule organization, *Z*-disc structure, and cell–cell adhesion. These structural networks showed a mixed pattern of up- and downregulation, suggesting dynamic remodeling of contractile architecture. Moreover, networks related to extracellular matrix organization, as well as complement and coagulation cascades, were **predominantly upregulated**, suggesting remodeling of the cardiac extracellular environment. Vesicular trafficking and intracellular transport pathways—including COPII-mediated transport, clathrin-coated vesicles, and the RISC complex—were also prominently represented, consistent with alterations in protein trafficking and post-transcriptional regulation.

### 3.4 Sex-specific baseline proteomic landscape in the heart

**Fig. 4:**
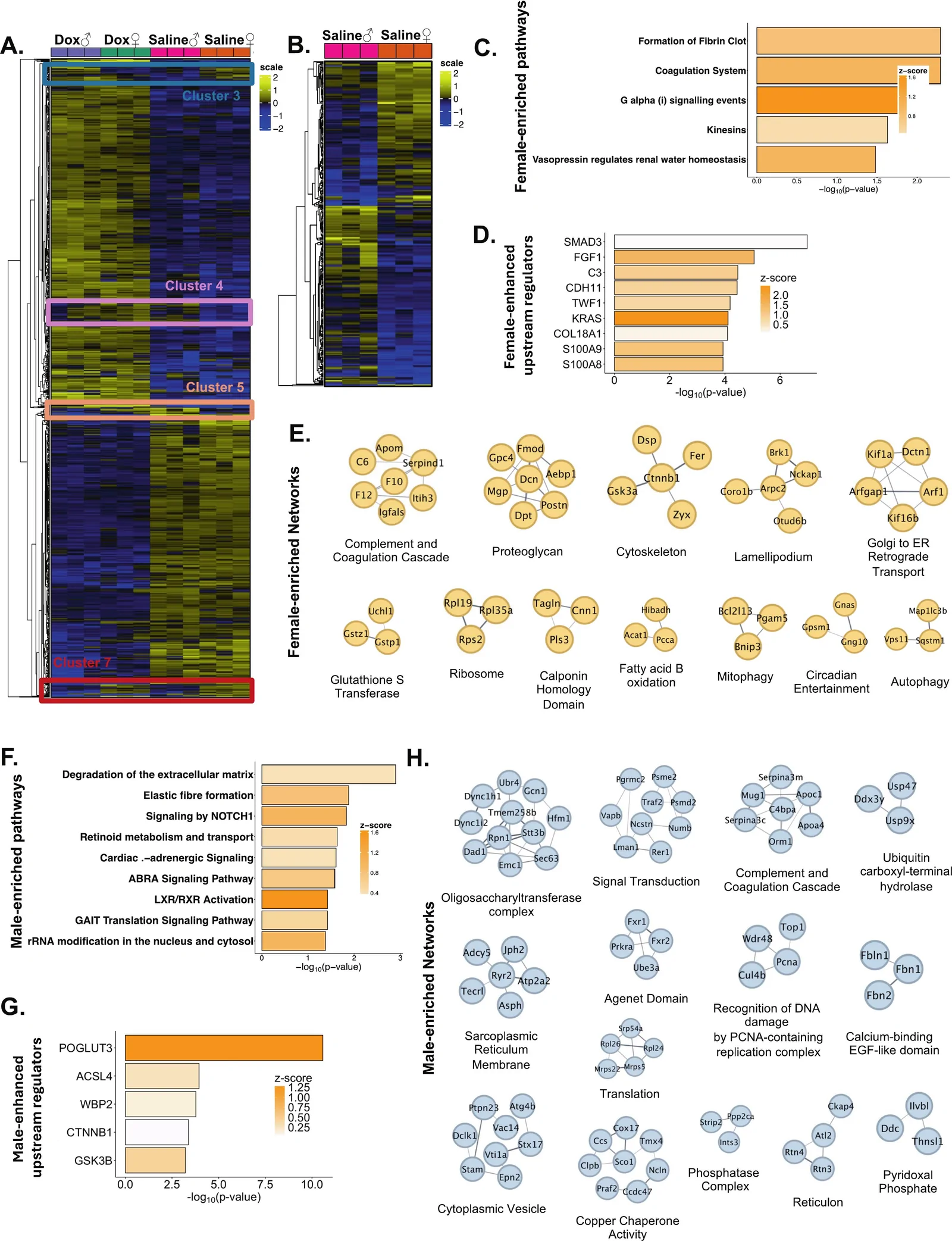
Baseline sexual dimorphism in the cardiac proteome, in the absence of doxorubicin: (A) Hierarchical clustering heatmap of significantly changing proteins (Male vs Female in Saline; two-way limma, p-value <0.05) highlighting four major clusters (Clusters 3, 4, 5 and 7) that distinguish male and female saline-treated hearts. (B) Heatmap of proteins exhibiting significant differences between female and male saline hearts. Out of 5184 proteins detected, 421 showed significant sex-based variation. (C) Ingenuity Pathway Analysis (IPA) reveals pathways inferred to be activated in female saline hearts (Fisher's exact test) compared to male saline hearts. (D) Upstream regulators inferred to be activated in female hearts compared to male saline hearts. (E) STRING network clustering of female-enriched proteins demonstrates coordinated enrichment in distinct biological modules. (F) IPA of male-enriched proteins identifies pathways inferred to be activated (Fisher's exact test) compared to female saline hearts. (G) Upstream regulators inferred to be activated in male hearts to female saline hearts. (H) STRING network analysis of male-enriched proteins reveals organization into distinct functional modules. *n* = 3 biologically independent animals per group (Male Saline, Female Saline). Each sample represents an individual rat heart.

Hierarchical clustering analysis revealed four major clusters (Clusters 3, 4, 5 and 7) that highlighted saline-treated female and male hearts differences (Fig. 4A). Of the 5184 proteins detected in the whole proteome dataset, 421 were significantly different between male and female saline hearts (pairwise comparison *p*-value <0.05, two-way limma), indicating baseline differences in protein abundance (Fig. 4B). IPA of female-enriched proteins identified pathways inferred to be activated in female saline treated hearts compared to male (Fisher's exact test), including fibrin clot formation and coagulation signaling, vasopressin-mediated water homeostasis, kinesin signaling, and G-alpha signaling (Fig. 4C). URA revealed multiple regulators inferred to be activated in female hearts compared to male hearts at baseline, including SMAD3, FGF1, C3, CDH11, TWF1, KRAS, COL18A1, S100A9, and S100A8, consistent with enhanced extracellular matrix remodeling, fibroblast activity, and immune-related signaling (Fig. 4D). Network-based annotation using Markov clustering and STRING database demonstrated coordinated enrichment of female-enriched proteins in biological modules including complement and coagulation cascades, proteoglycan components, cytoskeleton organization, fatty acid β-oxidation, mitophagy, circadian regulation, and autophagy pathways (Fig. 4E).

In male saline treated hearts pathways inferred to be activated compared to female hearts (Fisher's exact test) included extracellular matrix turnover, Notch and MAPK signaling, sarcoplasmic reticulum and membrane trafficking, retinoid metabolism, and mRNA regulation (Fig. 4F). Moreover, upstream regulators inferred to be activated encompassed POGLUT3, ACSL4, WBP2, CTNNB1, and GSK3B, consistent with structural remodeling, altered metabolic regulation, and stress associated signaling (Fig. 4G). Network-based annotation revealed biological modules related to signal transduction, complement and coagulation cascades, DNA damage recognition and repair, calcium regulation, ubiquitin-mediated proteolysis, and sarcoplasmic reticulum membrane dynamics (Fig. 4H). Together, these results demonstrate that even at baseline, the cardiac proteome exhibits sex-dependent differences prior to doxorubicin exposure.

### 3.5 Sex-dependent proteomic responses to doxorubicin in male versus female hearts

**Fig. 5:**
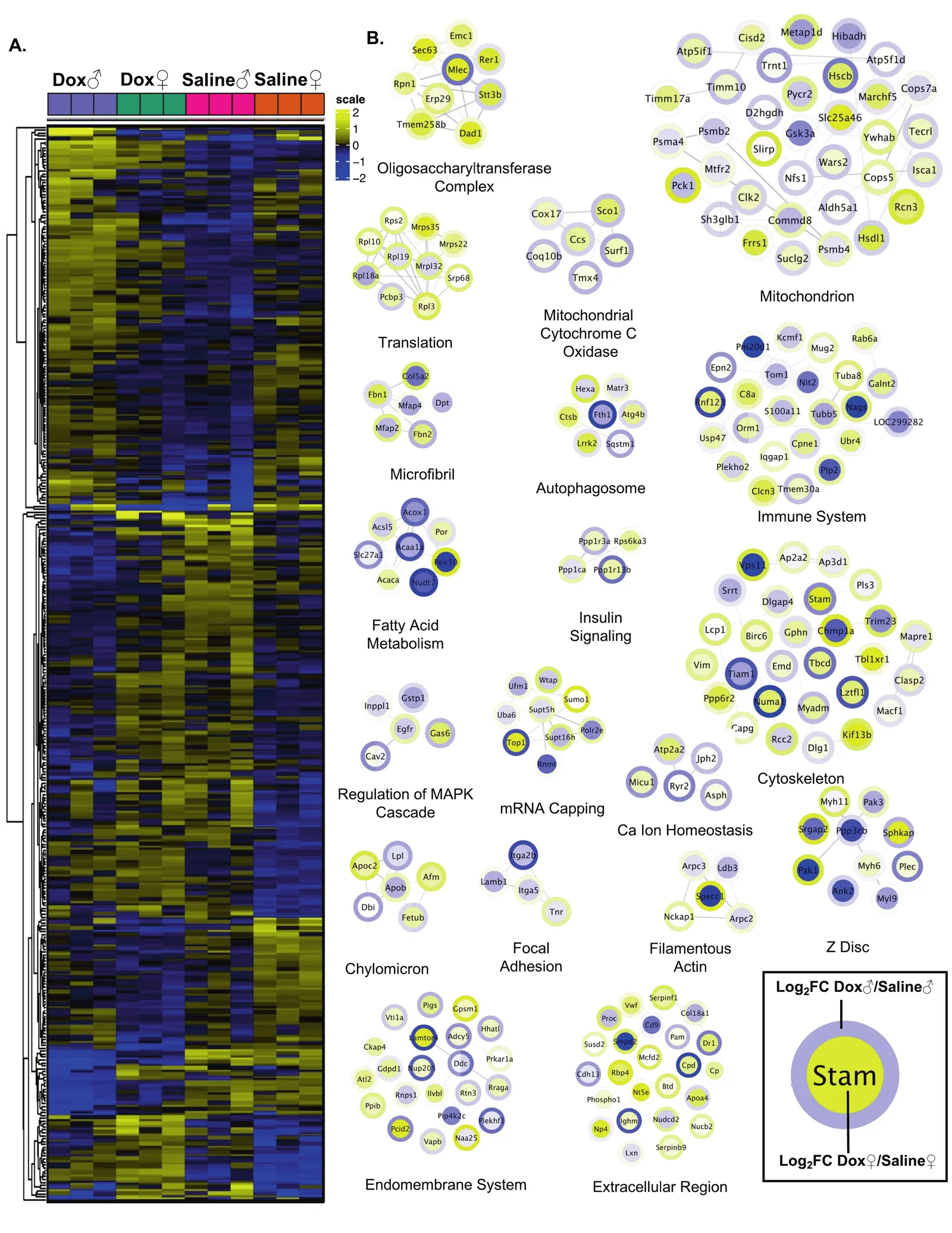
Sex-dependent proteomic responses to doxorubicin revealed by interaction-based network analysis. (A) Heat map with hierarchical clustering of proteins exhibiting a significant sex × treatment interaction (two-way limma, p-value <0.05), indicating proteins whose response to doxorubicin differs between females and males. (B) STRING network analysis of the same interaction-significant protein set shown in (A), highlighting functionally connected modules within the sex-dependent doxorubicin response. In each node, the inner circle represents the log₂ fold change (doxorubicin vs. saline) in females, while the outer circle represents the corresponding change in males. Yellow indicates upregulation and blue indicates downregulation in doxorubicin-treated animals. n = 3 biologically independent animals per group (Male Saline, Male Doxorubicin, Female Saline, Female Doxorubicin). Each sample represents an individual rat heart. (For interpretation of the references to colour in this figure legend, the reader is referred to the web version of this article.)

**Fig. 6:**
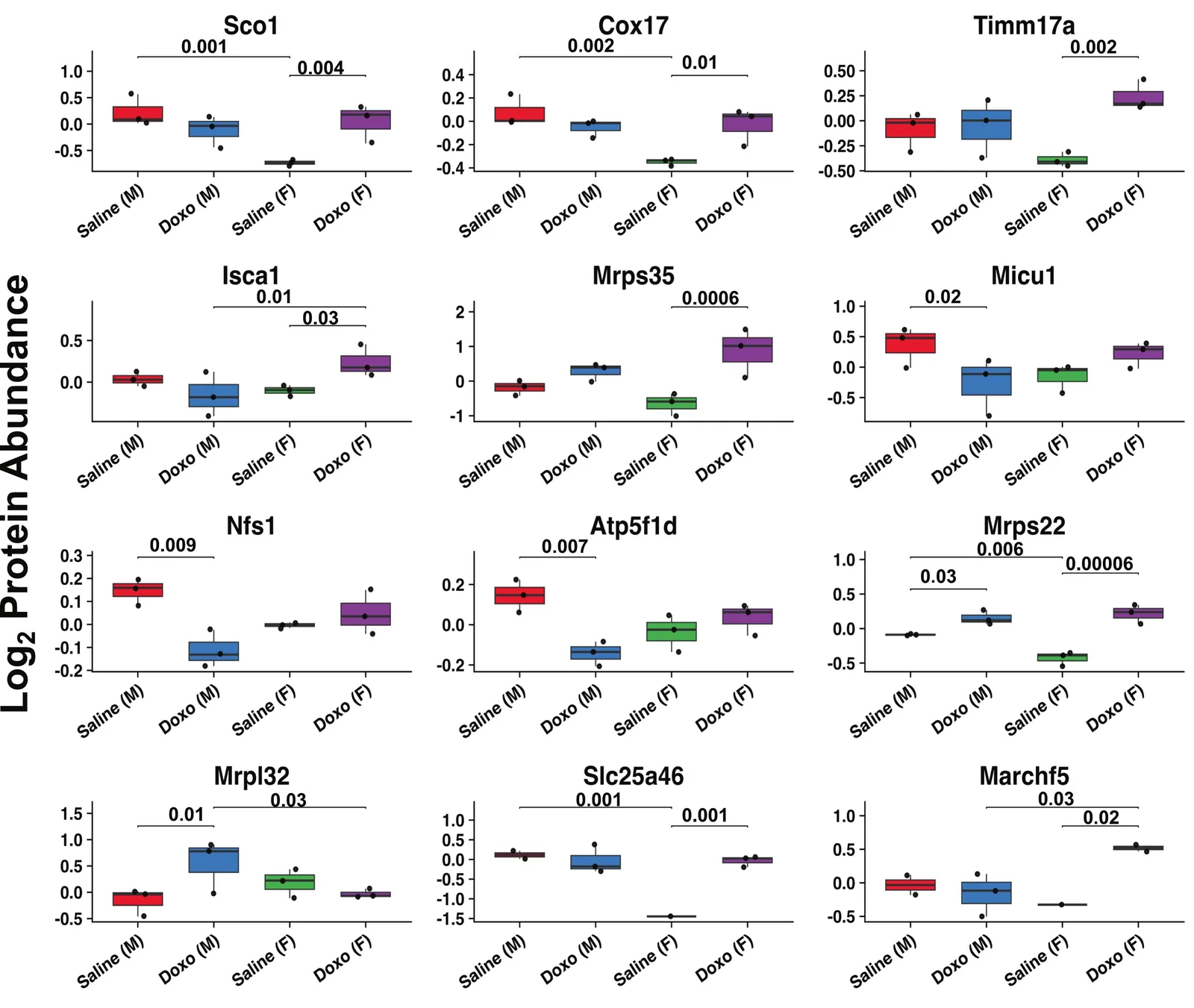
Box plots of mitochondrial–related proteins. Exhibiting a significant sex-by-treatment interaction (*p* < 0.05), identified by two-way limma analysis. Shown are normalized log₂ protein abundances across saline- and doxorubicin-treated male and female groups. Boxes represent the interquartile range (IQR) with the horizontal line indicating the median; individual data points are overlaid. *P* values correspond to pairwise contrasts extracted from the fitted two-way LIMMA model. n = 3 biologically independent animals per group (Male Saline, Male Doxorubicin, Female Saline, Female Doxorubicin). Each data point represents an individual rat heart.

**Fig. 7:**
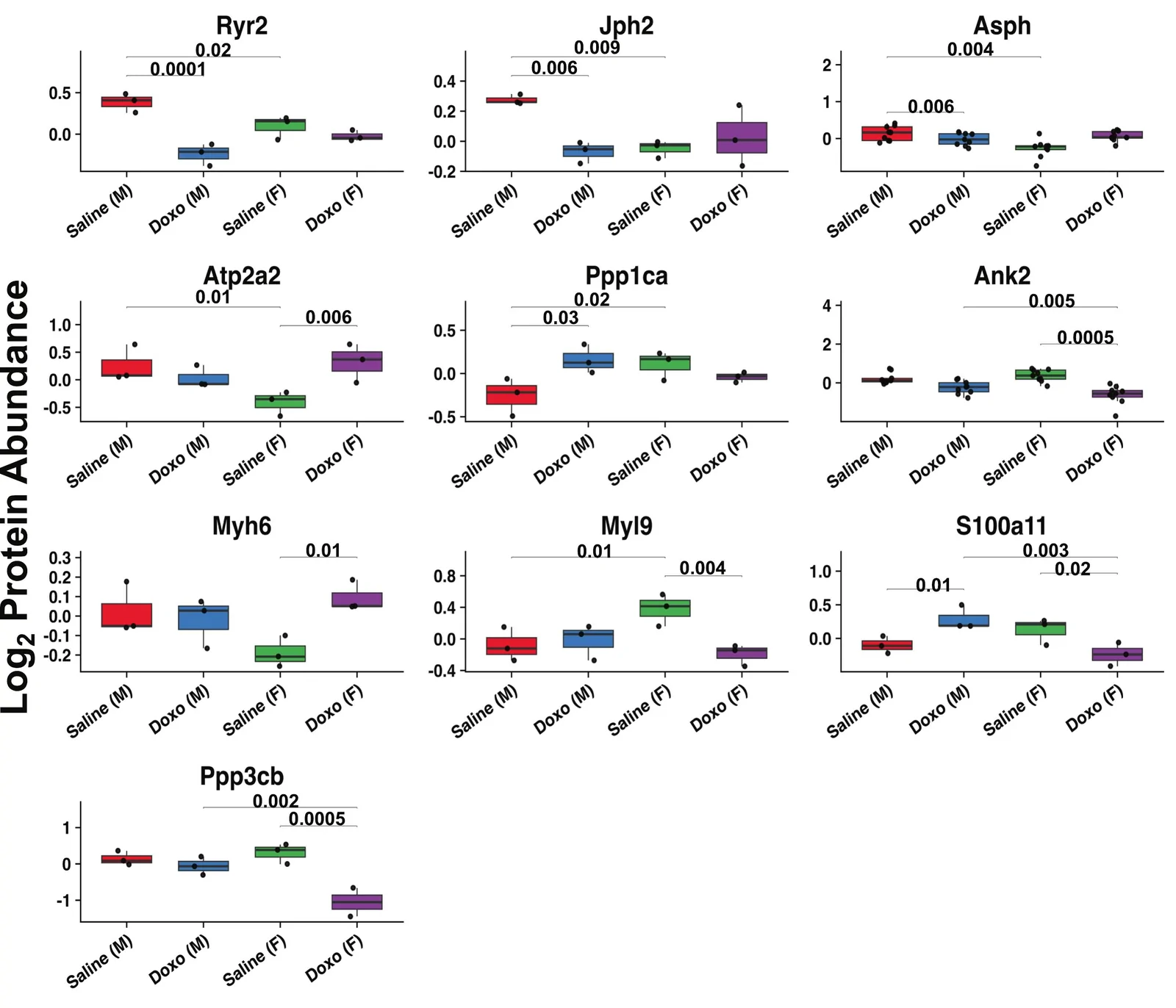
Box plots of excitation–contraction coupling proteins. Exhibiting a significant sex-by-treatment interaction (*p* < 0.05), identified by two-way limma analysis. Shown are normalized log₂ protein abundances across saline- and doxorubicin-treated male and female groups. Boxes represent the interquartile range (IQR) with the horizontal line indicating the median; individual data points are overlaid. P values correspond to pairwise contrasts extracted from the fitted two-way limma model. n = 3 biologically independent animals per group (Male Saline, Male Doxorubicin, Female Saline, Female Doxorubicin). Each data point represents an individual rat heart.

To identify proteins exhibiting sex-dependent responses to doxorubicin, we applied a two-way limma model incorporating sex, treatment, and their interaction. Filtering on the sex × treatment interaction term (*p*-value <0.05) identified 430 proteins whose response to doxorubicin differed significantly between males and females. Hierarchical clustering of these proteins revealed distinct response patterns across doxorubicin- and saline-treated hearts in both sexes **(**Fig. 5A**)**, suggesting coordinated but divergent proteomic trajectories. Among the interaction-significant proteins, 211 exhibited a greater doxorubicin-induced change in males, whereas 219 showed a greater response in females, relative to their respective saline controls. Network analysis of this protein set using STRING revealed multiple interconnected functional modules, including immune-related proteins, cytoskeletal organization, calcium ion homeostasis, fatty acid metabolism, subset of MAPK regulatory proteins, mitochondrial processes, and endomembrane system components **(**Fig. 5B**).** These proteins were selected based on a significant sex-by-treatment interaction (*p* < 0.05), reflecting sex-dependent responses to doxorubicin. Specifically, these proteins either exhibited opposite directional changes between females and males following treatment, or changed in the same direction in both sexes but with a significantly greater magnitude in one sex. In each node, the inner circle represents the log₂ fold change (doxorubicin vs. saline) in females, while the outer circle represents the corresponding change in males; yellow indicates upregulation and blue indicates downregulation in doxorubicin-treated animals. A subset of excitation–contraction and mitochondrial proteins exhibiting a significant sex-by-treatment interaction (p < 0.05) is shown in Fig. 6, Fig. 7 and represents candidate proteins for future mechanistic investigation.

## 4 Discussion

In this study, we performed large-scale data-independent acquisition (DIA) proteomics in a rat model of doxorubicin-induced cardiomyopathy with extended latency. We observed a more prominent gradual decline in fractional shortening in male rats treated with doxorubicin compared with females, suggesting greater functional decline in males at this timepoint. We performed proteomic analysis one week after the final injection to identify early molecular changes that occur before long-term functional decline, and to generate hypotheses about molecular differences that may contribute to later sex-specific outcomes.

Previous studies have shown that elevated cardiac uptake of a PET tracer targeting fibroblast activation protein α (FAP), can be detected as early as two weeks post-treatment in mice receiving a cumulative doxorubicin dose of 24 mg/kg, occurring before any functional deficits become apparent [31]. Additionally, early molecular markers, such as an increase in cardiac troponin levels, are detectable in some patients as early as the end of the first anthracycline administration cycle [5]. Consistent with these findings, our study revealed that doxorubicin treatment led to early proteomic remodeling as soon as one week after the last injection. This remodeling spanned multiple biological pathways, including stress-response mechanisms, mitochondrial function, contractile machinery, and metabolic processes—with a particular impact on fatty acid metabolism. Furthermore, analysis of the core sex-dependent proteins revealed that the differences between male and female responses did not arise from entirely distinct biological pathways, but rather from differences within the same networks that define the global doxorubicin response. In the discussion that follows, we first address the global trends of doxorubicininduced cardiac toxicity, then turn to the sex-dependent differences.

### 4.1 Mitochondrial protein dysregulation and oxidative phosphorylation impairment

A prominent finding was the **alteration of numerous mitochondrial and metabolic proteins** in DOX-treated hearts. These changes suggest impaired mitochondrial function and reduced energy production capacity. Key alterations included: multiple complex I subunits (Ndufa and Ndufs families) along with assembly/stability factors such as **Foxred1** and **Acad9** that were downregulated, which would be consistent with impaired complex I biogenesis and electron transfer capacity. Decrease abundance of components of complex III (**Uqcrc1**) further supports compromised oxidative phosphorylation. Downregulation of accessory proteins critical for inner membrane structure and cristae organization (e.g., **Apoo)** is consistent with disrupted mitochondrial structural integrity. Enzymes involved in mitochondrial NADPH generation, including **Nnt** and **Nadk2**, were also decreased. Together, these suggest an **impairment of oxidative phosphorylation.** These combined effects may contribute to mitochondrial swelling, cristae disruption, impaired energy metabolism, and opening of the mitochondrial permeability transition pore, ultimately resulting in cardiomyocyte death [2], [32]. These proteomic findings are consistent with prior functional studies demonstrating impaired mitochondrial respiration, reduced membrane potential, and disrupted NADH redox state in doxorubicin-treated hearts [33], [34], [35].

### 4.2 Metabolic shifts and regulatory changes

While doxorubicin-induced impairment of glucose utilization and fatty acid oxidation has been previously reported [4], our unbiased proteomic approach captures this metabolic shift as part of a broader, coordinated remodeling occurring as early as one week after the final dose. Consistent with the observed mitochondrial protein changes, we found that proteins involved in fatty acid β-oxidation, glucose metabolism (glycolysis), and pyruvate metabolism were prominently affected. These changes are consistent with a shift toward inefficient energy production that could predispose the heart to long-term compromised cardiac contractility. In essence, the heart's energy substrate preference appears to shift away from fatty acids toward glucose, but without efficient ATP generation. Supporting this interpretation, pathway analysis revealed significant downregulation of lipid metabolic pathways, including fatty acyl-CoA biosynthesis. Upstream regulator analysis provided additional insight into this metabolic shift. Key metabolic regulators were inhibited or activated in a manner consistent with the observed proteomic changes. For instance, RXRA and LPL, crucial drivers of fatty acid oxidation and mitochondrial gene expression [36], [37], were predicted to be inhibited in DOX-treated hearts. Concurrently RAC1 was inferred to be activated, which suggests an amplification of ROS production [38]. Together, these regulator-level findings extend prior reports of impaired substrate utilization by identifying specific upstream drivers that may coordinate this early metabolic remodeling, offering a mechanistic link not previously described at the proteomic level.

Cytoskeletal remodeling

Beyond metabolic changes, DOX also provoked significant remodeling of the cytoskeletal and stress-response networks in the heart. We observed decreased abundance of several structural proteins integral to the cardiomyocyte contractile apparatus, consistent with altered structural integrity of cardiac muscle fibers. This is consistent with prior reports creating energetic deficits that may compromise contractile function [4]. At the pathway level, this remodeling was reflected by activation of actin cytoskeleton signaling alongside suppression of calcium signaling, suggesting dysregulated excitation–contraction coupling in DOX-treated hearts.

### 4.3 Proteostasis and stress responses

In parallel, proteomic network analysis revealed substantial reprogramming of cardiac proteostasis. Several network modules were enriched for proteins involved in RNA splicing, protein translation, and protein turnover, suggesting the heart undergoes extensive protein homeostasis adjustments under DOX-induced injury. Among these, RNA splicing emerged as a prominent feature of the network. Dysregulation of splicing factor expression has previously been reported in failing hearts, leading to disrupted alternative splicing and impaired cardiac function [39]. In our network analysis, spliceosome proteins were detected as a module, including Srsf1, which was downregulated. Notably, Srsf1 overexpression has been shown to improve cardiac function and reduce infarct size [40]. Importantly, among the downregulated structural proteins was Mylk3, the primary cardiac myosin light chain kinase responsible for tuning actomyosin crossbridge kinetics to heartbeat frequency. Cardiomyocyte-specific ablation of Mylk3 is sufficient to cause heart failure [41], suggesting that its downregulation in DOX-treated hearts may directly contribute to the contractile dysfunction observed in this model.

Furthermore, we identified a large, coordinated network of proteins associated with general cellular stress responses. This included many chaperones and co-chaperones involved in protein folding, cytokine signaling, and neutrophil degranulation proteins. The enrichment of these proteins may suggest an activated defense mechanism attempting to mitigate DOX-induced damage. Prior studies have also implicated several additional mechanisms in DOX-induced cardiac dysfunction, such as ferroptosis [42], inflammatory cell infiltration [8], calcium dysregulation, endoplasmic reticulum stress, and altered MAPK signaling as additional contributors to progressive cardiac dysfunction [43], [44].

### 4.4 Sex-based differences at baseline and under doxorubicin

One of the key observations from our analysis is that male and female hearts exhibit distinct proteomic profiles even under baseline conditions (saline-treated controls). Upstream regulator analysis at baseline revealed that female hearts were enriched for regulators related to extracellular matrix remodeling (SMAD3, CDH11, COL18A1), inflammation (C3, S100A8, S100A9), cytoskeletal remodeling (TWF1), and stress-adaptive signaling pathways (FGF1, KRAS). In contrast, male hearts showed greater baseline enrichment of regulators tied to protein modification (POGLUT3, WBP2), lipid metabolism and ferroptosis pathway (ACSL4), and Wnt/β-catenin–mediated structural and growth signaling (CTNNB1, GSK3B). These intrinsic baseline differences may establish sex-specific regulatory states that influence how each sex responds to cardiac stress.

Upon doxorubicin exposure, male rats exhibited an earlier decline in cardiac function compared to females. By 7 months, males reached fractional shortening (FS) below 30%, whereas females required a longer duration to reach a comparable level of dysfunction. In our sex-stratified analysis of DOX effects, we identified proteins that changed significantly in one sex compared to the other (DOX vs. saline in males versus DOX vs. saline in females). These sex-differential proteins were mostly involved in calcium-handling proteins, **mitochondrial function,** fatty **acid metabolism, and immune responses**. Notably, proteins involved in cytoskeletal organization were more strongly altered in males following doxorubicin treatment, with the majority showing increased abundance, including Myh11, Myl9, Tagln, Tubb5, Tuba8, Pls3, Mapre1, and Capg.

While prior studies have implicated mitochondrial Ca^2+^ dysregulation and impaired energetics in doxorubicin cardiotoxicity, our data reveal a coordinated, female-specific upregulation of mitochondrial proteins involved in Complex IV assembly (Sco1, Cox17), protein import (Timm17A), Fe—S cluster maturation (Isca1), and mitochondrial translation machinery (Mrps35). Importantly, none of these proteins changed significantly in DOX-treated males relative to male controls — the response was confined to females. This sex-specific pattern is precisely what the sex×treatment interaction term in our two-way limma framework is designed to capture. In contrast, the reduction of Micu1 in doxorubicin-treated males may further contribute to calcium dysregulation, as loss of this mitochondrial calcium gatekeeper has been shown to increase calcium influx and sensitize mitochondria to calcium overload injury [45]. Males exhibited reduced Fe—S cluster biogenesis (Nfs1), and decreased ATP synthase subunit Atp5f1d, consistent with impaired energetic reserve and heightened sensitivity to Ca^2+^ and ROS-mediated mitochondrial stress.These findings are particularly interesting given the role of Fe—S cluster regulation in ferroptosis-associated cardiomyocyte injury. H₂S has been shown to attenuate doxorubicin-induced cardiotoxicity by inhibiting ferroptosis through OPA3 S‑sulfuration and NFS1-dependent regulation of Fe—S biology [46]. **(**Fig. 6**).**

These mitochondrial protein changes were accompanied by alterations in calcium handling and excitation–contraction coupling proteins in males, raising the possibility of a mechanistic link to the greater functional decline observed in males. Increased Pp1 activity has been reported to promote dephosphorylation of phospholamban [47], thereby inhibiting SERCA2a-mediated calcium reuptake into the sarcoplasmic reticulum, while RyR2 serves as the primary channel for calcium release from sarcoplasmic reticulum. The increased Ppp1ca and reduced abundance of RyR2 therefore may be consistent with of impaired excitation–contraction coupling and negative regulation of cardiac function [48], [49]. The concurrent Jph2 decrease is notable, as Jph2 maintains SR/T-tubule junctional integrity and its reduction is consistently observed in heart failure [50]. In contrast, females displayed increased Atp2a2 (SERCA2a) and Myh6, consistent with changes that may support Ca^2+^ reuptake and contractility. Female rats showed significant downregulation of Ppp3cb (calcineurin). This sex-specific difference in calcineurin expression is of interest, as calcineurin-NFAT signaling has been reported to drive pathological hypertrophy [8], fibrosis, and cardiac remodeling [43], [51] (Fig. 7). Collectively, these findings support previous observations that male rats experience marked disruption of contractile and cytoskeletal integrity following anthracycline exposure, manifesting as severe reductions in LVEF, pulmonary edema, ascites, and myocardial fibrosis. In contrast, female showed relative preservation of mechanical function, consistent with prior observations in rat models of doxorubicin cardiotoxicity [52] Moulin et al. demonstrated that mitochondrial dysfunction is involved in early cardiotoxicity in males, characterized by reduced Pgc-1β expression, impaired fatty acid oxidation and respiration, and decreased mitochondrial DNA content [44], [52]. More recently, Abdelgawad et al. applied proteomics to a mouse model of DOX cardiotoxicity and identified sex-specific proteomic responses, with male mice showing greater cardiac dysfunction [53]; however, that study was limited to 2461 proteins and analyzed each sex independently without a formal sex×treatment interaction framework. Our study extends these findings using DIA proteomics across 5184 proteins with two-way limma, providing substantially greater depth and statistical resolution of sex-specific DOX responses.

### 4.5 Limitations

Several limitations of this study should be acknowledged. First, proteomic profiling was performed at a single timepoint — one week after the final doxorubicin injection — which captures an early snapshot of the molecular response but does not allow us to distinguish whether the sex-specific proteomic changes represent qualitatively distinct responses or differences in the temporal trajectory of a shared response. Longitudinal proteomic profiling across multiple timepoints would be needed to resolve this question, and we cannot confirm which early proteomic changes persist to drive long-term functional decline. Second, echocardiographic assessment was limited to fractional shortening, and diastolic function — recognized as an early indicator of anthracycline cardiotoxicity — was not evaluated; a more comprehensive functional characterization would strengthen future studies. Finally, while this rat model recapitulates key features of doxorubicin-induced cardiomyopathy, extrapolation of these findings to human cardiotoxicity should be done cautiously given interspecies differences in cardiac physiology and drug metabolism.

## 5 Conclusion

Taken together, the proteins showing sex-specific responses to DOX — whether in mitochondrial function, calcium handling, cytoskeletal organization, or metabolism — belong to the same core biological pathways that define the overall cardiac response to doxorubicin. Sex does not appear to engage an entirely distinct molecular program; rather, it modulates how individual proteins within these shared networks are regulated. Males and females mount responses through the same fundamental pathways, but the direction, magnitude, and pattern of protein-level changes differ between sexes. For example, female hearts showed upregulation of the mitochondrial proteins Sco1, Cox17, and Atp2a2, potentially reflecting preserved energetics and Ca^2+^ handling, along with reduced calcineurin (Ppp3cb), whereas male hearts showed reduced Micu1 and RyR2 alongside increased Ppp1ca, consistent with impaired mitochondrial function and excitation–contraction coupling.These proteomic patterns represent candidate molecular targets that warrant functional validation in future longitudinal and mechanistic studies, and suggest that sex acts as a modifier of the DOX-induced cardiac proteomic response, contributing to the observed differences in the extent and trajectory of cardiac remodeling.

## CRediT authorship contribution statement

**Sogol Sedighi:** Writing – review & editing, Writing – original draft, Formal analysis, Data curation, Conceptualization. **Lijun Chen:** Formal analysis, Data curation, Conceptualization. **Yuefen Wang:** Formal analysis, Data curation. **Kaui Lebarbenchon:** Formal analysis, Data curation. **Jena Yi:** Formal analysis, Data curation. **Tanvi Menghani:** Formal analysis, Data curation. **Firasat Ali Shah:** Formal analysis, Data curation. **Sam Yang:** Formal analysis, Data curation. **Harumi Saeki:** Formal analysis, Data curation. **Tomoaki Ito:** Funding acquisition, Formal analysis, Data curation. **Hajime Orita:** Formal analysis, Data curation. **Hui Zhang:** Supervision, Software, Resources, Formal analysis, Data curation. **Brian O’Rourke:** Writing – review & editing, Validation, Supervision, Conceptualization. **D. Brian Foster:** Writing – review & editing, Writing – original draft, Visualization, Validation, Supervision, Resources, Funding acquisition, Data curation, Conceptualization, Project administration. **Kathleen L. Gabrielson:** Writing – review & editing, Writing – original draft, Visualization, Validation, Supervision, Resources, Methodology, Funding acquisition, Conceptualization.

## Funding

This study was supported in part by a Grant-in-Aid from MEXTSupported Program for the Strategic Research Foundation at Private Universities, 2015–19. T. Ito, H. Saeki. KG was supported by NIH R01HL156224, R21HL156224. DBF was supported by NIH R01HL134821, R01HL164478, USAMRAA HT94252410277, AHA 18TPA34170575, and the 10.13039/100009364Saving Tiny Hearts Society.

## Declaration of competing interest

The authors declare that they have no competing interests.

## Data Availability

The MS proteomics raw data (.raw) have been deposited to the ProteomeXchange Consortium (http://www.proteomexchange.org/; [54])via the PRIDE partner repository [54] with the data set identifier PXD076815. All code used for normalization, statistical analysis, PCA, and heat map generation is publicly available at: https://github.com/Frostman300/dox-upload.

## Appendix A Supplementary data

Supplementary Fig. 1 Representative expression patterns of proteins within smaller clusters identified in the heatmap of the global doxorubicin-responsive proteome. Shown are Clusters 3–7 extracted from the hierarchical clustering heatmap presented in Fig. 2. These clusters comprise proteins identified as significant in the global (omnibus) F-test from a two-way limma model incorporating sex and treatment as factors. Together, these clusters illustrate distinct expression trajectories within the broader doxorubicin-associated proteomic response.(A) Cluster 3, proteins exhibiting higher abundance in doxorubicin-treated male hearts compared with doxorubicin-treated female hearts, accompanied by an opposite baseline sex bias under saline conditions.(B) Cluster 4, proteins exhibiting higher abundance in saline-treated male hearts compared with saline-treated female hearts.(C) Cluster 5, proteins exhibiting higher abundance in saline-treated males compared with saline-treated females, with differential responses to doxorubicin treatment.(D) Cluster 6, proteins displaying divergent responses to doxorubicin treatment between experimental groups. (E) Cluster 7, proteins exhibiting consistently higher abundance in female hearts under both saline and doxorubicin conditions. *n* = 3 biologically independent animals per group (Male Saline, Male Doxorubicin, Female Saline, Female Doxorubicin). Each data point represents an individual rat heart.

Supplementary Table 1 Detailed protein quantification, fold changes across experimental groups, and statistical significance calculated using a two-way limma model.

## References

[1] Bernstein D. (2018). Anthracycline cardiotoxicity: worrisome enough to have you quaking?. Circ Res.

[2] Wallace K.B., Sardão V.A., Oliveira P.J. (2020). Mitochondrial determinants of doxorubicin-induced cardiomyopathy. Circ Res.

[3] Jenkins G.R. (2016). Sex-related differential susceptibility to doxorubicin-induced cardiotoxicity in B6C3F1 mice. Toxicol Appl Pharmacol.

[4] Qing G., Huang C., Pei J., Peng B. (2025). Alteration of cardiac energetics and mitochondrial function in doxorubicin-induced cardiotoxicity: Molecular mechanism and prospective implications. Int J Mol Med.

[5] Vejpongsa P., Yeh E.T.H. (2014). Prevention of anthracycline-induced cardiotoxicity: challenges and opportunities. J Am Coll Cardiol.

[6] Rawat P.S., Jaiswal A., Khurana A., Bhatti J.S., Navik U. (2021). Doxorubicin-induced cardiotoxicity: an update on the molecular mechanism and novel therapeutic strategies for effective management. Biomed Pharmacother.

[7] Díaz-Guerra A. (2024). Anthracycline cardiotoxicity induces progressive changes in myocardial metabolism and mitochondrial quality control: novel therapeutic target. JACC: CardioOncol.

[8] Sun Y., Xiao L., Chen L., Wang X. (2025). Doxorubicin-induced cardiac remodeling: mechanisms and mitigation strategies. Cardiovasc Drugs Ther.

[9] Wilcox N.S. (2022). Sex-specific cardiovascular risks of cancer and its therapies. Circ Res.

[10] Lipshultz S.E. (2019). Cardiomyopathy in children: classification and diagnosis: a scientific statement from the American Heart Association. Circulation.

[11] He H. (2025). Sex-based differences in mitochondrial activity and cellular calcium signaling in isolated mice cardiomyocytes intervened by doxorubicin. Biochem Biophys Res Commun.

[12] Desai V.G. (2023). Potential role of the apelin-APJ pathway in sex-related differential cardiotoxicity induced by doxorubicin in mice. J Appl Toxicol.

[13] Rattanasopa C. (2019). Estrogen but not testosterone preserves myofilament function from doxorubicin-induced cardiotoxicity by reducing oxidative modifications. Am J Physiol Heart Circ Physiol.

[14] Yuan Y. (2020). Exploration the mechanism of doxorubicin-induced heart failure in rats by integration of proteomics and metabolomics data. Front Pharmacol.

[15] Wachtman L.M., Browning M.D., Bedja D., Pin S., Gabrielson K.L. (2006). Validation of the use of long-term indwelling jugular catheters in a rat model of cardiotoxicity. J Am Assoc Lab Anim Sci.

[16] Andreuzzi E. (2018). Loss of Multimerin-2 and EMILIN-2 expression in gastric cancer associate with altered angiogenesis. Int J Mol Sci.

[17] Gabrielson K. (2007). Heat shock protein 90 and ErbB2 in the cardiac response to doxorubicin injury. Cancer Res.

[18] Mertins P. (2018). Reproducible workflow for multiplexed deep-scale proteome and phosphoproteome analysis of tumor tissues by liquid chromatography-mass spectrometry. Nat Protoc.

[19] Bruderer R. (2017). Optimization of experimental parameters in data-independent mass spectrometry significantly increases depth and reproducibility of results. Mol Cell Proteomics.

[20] Escher C. (2012). Using iRT, a normalized retention time for more targeted measurement of peptides. Proteomics.

[21] Reiter L. (2011). mProphet: automated data processing and statistical validation for large-scale SRM experiments. Nat Methods.

[22] Herbrich S.M. (2013). Statistical inference from multiple iTRAQ experiments without using common reference standards. J Proteome Res.

[23] Foster D.B. (2016). Integrated omic analysis of a guinea pig model of heart failure and sudden cardiac death. J Proteome Res.

[24] Smyth G.K. (2004). Linear models and empirical bayes methods for assessing differential expression in microarray experiments. Stat Appl Genet Mol Biol.

[25] Storey J.D., Tibshirani R. (2003). Statistical significance for genomewide studies. Proc Natl Acad Sci.

[26] Storey J.D. (2002). A direct approach to false discovery rates. J R Stat Soc Ser B Stat Methodol.

[27] Doncheva N.T. (2023). Cytoscape stringApp 2.0: analysis and visualization of heterogeneous biological networks. J Proteome Res.

[28] Shannon P. (2003). Cytoscape: a software environment for integrated models of biomolecular interaction networks. Genome Res.

[29] Van Dongen S. (2008). Graph clustering via a discrete uncoupling process. SIAM J Matrix Anal Appl.

[30] Utriainen M., Morris J.H. (2023). clusterMaker2: a major update to clusterMaker, a multi-algorithm clustering app for Cytoscape. BMC Bioinform.

[31] Lee C.H. (2025). FAP PET identifies earlycardiac molecular changesinduced by doxorubicin chemotherapy. JCI Insight.

[32] Murphy E. (2016). Mitochondrial function, biology, and role in disease: a scientific statement from the American Heart Association. Circ Res.

[33] Oliveira P.J., Santos M.S., Wallace K.B. (2006). Doxorubicin-induced thiol-dependent alteration of cardiac mitochondrial permeability transition and respiration. Biochemistry (Mosc).

[34] Montaigne D. (2010). Stabilization of mitochondrial membrane potential prevents doxorubicin-induced cardiotoxicity in isolated rat heart. Toxicol Appl Pharmacol.

[35] Abdullah C.S. (2022). Monitoring mitochondrial morphology and respiration in doxorubicin-induced cardiomyopathy. Methods Mol Biol.

[36] Osorio J.C. (2002). Impaired myocardial fatty acid oxidation and reduced protein expression of retinoid X Receptor-α in pacing-induced heart failure. Circulation.

[37] Shang R., Rodrigues B. (2021). Lipoprotein lipase and its delivery of fatty acids to the heart. Biomolecules.

[38] Li J. (2010). Deficiency of rac1 blocks NADPH oxidase activation, inhibits endoplasmic reticulum stress, and reduces myocardial remodeling in a mouse model of type 1 diabetes. Diabetes.

[39] Giménez-Escamilla I. (2024). Transcriptomic alterations in spliceosome components in advanced heart failure: status of cardiac-specific alternative splicing factors. Int J Mol Sci.

[40] Xie Y. (2024). Splicing factor SRSF1 attenuates cardiomyocytes apoptosis via regulating alternative splicing of Bcl2L12. Cell Biosci.

[41] Chang A.N., Kamm K.E., Stull J.T. (2016). Role of myosin light chain phosphatase in cardiac physiology and pathophysiology. J Mol Cell Cardiol.

[42] Li Y. Robert, Traore K., Zhu H. (2024). Novel molecular mechanisms of doxorubicin cardiotoxicity: latest leading-edge advances and clinical implications. Mol Cell Biochem.

[43] Narezkina A., Narayan H.K., Zemljic-Harpf A.E. (2021). Molecular mechanisms of anthracycline cardiovascular toxicity. Clin Sci (Lond).

[44] Ludke A., Akolkar G., Ayyappan P., Sharma A.K., Singal P.K. (2017). Time course of changes in oxidative stress and stress-induced proteins in cardiomyocytes exposed to doxorubicin and prevention by vitamin C. PLoS One.

[45] Hasan P. (2024). MICU1 and MICU2 control mitochondrial calcium signaling in the mammalian heart. Proc Natl Acad Sci USA.

[46] Wang Y. (2023). Hydrogen sulfide alleviates mitochondrial damage and ferroptosis by regulating OPA3–NFS1 axis in doxorubicin-induced cardiotoxicity. Cell Signal.

[47] Shintani-Ishida K., Yoshida K. (2011). Ischemia induces phospholamban dephosphorylation via activation of calcineurin, PKC-α, and protein phosphatase 1, thereby inducing calcium overload in reperfusion. Biochim Biophys Acta.

[48] Bround M.J. (2016). Cardiac Ryanodine Receptor (Ryr2)-mediated calcium signals specifically promote glucose oxidation via pyruvate dehydrogenase. J Biol Chem.

[49] Carr A.N. (2002). Type 1 phosphatase, a negative regulator of cardiac function. Mol Cell Biol.

[50] Guo A. (2014). Overexpression of junctophilin-2 does not enhance baseline function but attenuates heart failure development after cardiac stress. Proc Natl Acad Sci USA.

[51] Parra V., Rothermel B.A. (2017). Calcineurin signaling in the heart: the importance of time and place. J Mol Cell Cardiol.

[52] Moulin M. (2015). Sexual dimorphism of doxorubicin-mediated cardiotoxicity. Circ Heart Fail.

[53] Abdelgawad I.Y. (2024). Sex-related differences in delayed doxorubicin-induced cardiac dysfunction in C57BL/6 mice. Arch Toxicol.

[54] Deutsch E.W. (2023). The ProteomeXchange consortium at 10 years: 2023 update. Nucleic Acids Res.

