# Supplementary material for: Early cardiac proteomic profiling in a long-latency doxorubicin cardiomyopathy model exhibits sexual dimorphism for cardioprotection
Source: J Mol Cell Cardiol Plus. 2026 Aug 15;17:100861. doi: 10.1016/j.jmccpl.2026.100861 (PMC13521005; doi:10.1016/j.jmccpl.2026.100861)
Supplement: Supplementary file 1 [file mmc1.pdf]

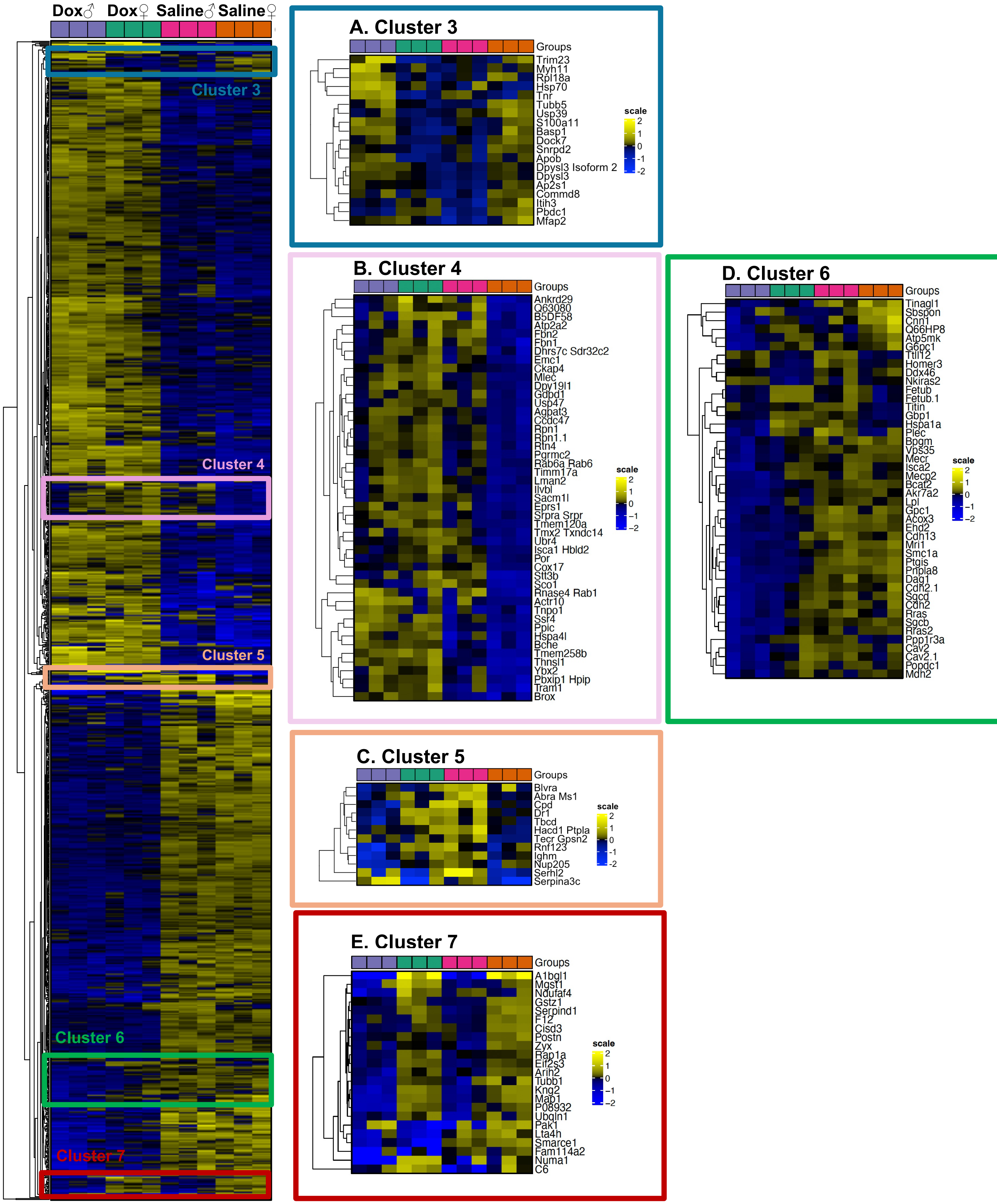

Dox♂ Dox♀ Saline♂ Saline♀
Cluster 3
Cluster 4
Cluster 5
Cluster 6
Cluster 7
A. Cluster 3
Groups
Trim23
Myh11
Rpl18a
Hsp70
Tnr
Tubb5
Usp39
S100a11
Basp1
Dock7
Snrpd2
Apob
Dpysl3 Isoform 2
Dpysl3
Ap2s1
Commd8
Itih3
Pbdc1
Mfap2
scale
B. Cluster 4
Ankrd29
Q63080
B5DF58
Atp2a2
Fbn2
Fbn1
Dhrs7c Sdr32c2
Emc1
Ckap4
Mlec
Dpy19l1
Gdpd1
Usp47
Agpat3
Ccdc47
Rpn1
Rpn1.1
Rtn4
Pgrmc2
Rab6a Rab6
Timm17a
Lman2
Ilvbl
Sacm1l
Eprs1
Srpra Srpr
Tmem120a
Tmx2 Txndc14
Ubr4
Isca1 Hbld2
Por
Cox17
Stt3b
Sco1
Rnase4 Rab1
Actr10
Tnpo1
Ssr4
Ppic
Hspa4l
Bche
Tmem258b
Thnsl1
Ybx2
Pbxip1 Hpip
Tram1
Brox
D. Cluster 6
Tinagl1
Sbspon
Cnn1
Q66HP8
Atp5mk
G6pc1
Ttll12
Homer3
Ddx46
Nkiras2
Fetub
Fetub.1
Titin
Gbp1
Hspa1a
Plec
Bpgm
Vps35
Mecr
Isca2
Mecp2
Bcat2
Akr7a2
Lpl
Gpc1
Acox3
Ehd2
Cdh13
Mri1
Smc1a
Ptgis
Pnpla8
Dag1
Cdh2.1
Sgcd
Cdh2
Rras
Sgcb
Rras2
Ppp1r3a
Cav2
Cav2.1
Popdc1
Mdh2
C. Cluster 5
Blvra
Abra Ms1
Cpd
Dr1
Tbcd
Hacd1 Ptpla
Tecr Gpsn2
Rnf123
Ighm
Nup205
Serhl2
Serpina3c
E. Cluster 7
A1bg1
Mgst1
Ndufaf4
Gstz1
Serpind1
F12
Cisd3
Postn
Zyx
Rap1a
Eif2s3
Arih2
Tubb1
Kng2
Map1
P08932
Ubqln1
Pak1
Lta4h
Smarce1
Fam114a2
Numa1
C6
